# From CBASS and Thoeris to Panoptes: Phage sponges and the inversion of bacterial immunity

**DOI:** 10.1371/journal.ppat.1014717

**Published:** 2026-10-27

**Authors:** Ruoqi Zheng, Leiliang Zhang

**Affiliations:** 1 Department of Infectious Diseases, Shandong Provincial Hospital Affiliated to Shandong First Medical University, Jinan, Shandong, China; 2 Department of Pathogen Biology, School of Clinical and Basic Medical Sciences, Shandong First Medical University & Shandong Academy of Medical Sciences, Jinan, Shandong, China; University of California, San Francisco, UNITED STATES OF AMERICA

## Introduction

Bacteria and bacteriophages have been engaged in an evolutionary arms race for billions of years. As the planet’s most abundant biological entities, phages outnumber bacteria roughly ten to one [1], driving constant selection for bacterial defense. In turn, bacteria have evolved diverse immune systems to detect and repel phage infection, while phages have devised equally elaborate countermeasures. This perpetual conflict has forged intricate molecular weaponry on both sides.

Nucleotide signaling is central to this molecular conflict. Bacteria use small diffusible nucleotides to report infection and coordinate immunity [2], while phages have evolved mechanisms to intercept or disrupt these signals [3–9]. The recent discovery of the Panoptes system reveals a surprising twist [10,11]. Bacteria take the molecules that phages seek to sequester and turn them into a self-destruct signal. This inverted logic extends our understanding of bacterial immunity and offers a new perspective on host-pathogen interactions.

## Bacterial weapons: CBASS, Thoeris, and other nucleotide-based defense systems

Several bacterial defense systems rely on nucleotide second messengers for immune signaling, including the cyclic oligonucleotide-based antiphage signaling system (CBASS), Thoeris, Pycsar, and type III CRISPR-Cas [2]. This pearl discusses these systems with a particular focus on CBASS and Thoeris, as they are central to the phage–sponge arms race that ultimately led to the discovery of Panoptes. Both CBASS and Thoeris operate through the production of nucleotide second messengers (Fig 1A). CBASS uses cyclic nucleotide signals [2], whereas Thoeris generates NAD^+^-derived signals that include both cyclic molecules, such as 1''-3' gcADPR [12] and N7-cADPR [6], and linear molecules, such as His-ADPR [13]. Sensor proteins detect phage infection, synthesize nucleotide signals, and activate downstream effectors, which in turn trigger defense responses including cell death or growth arrest.

**Fig 1:**
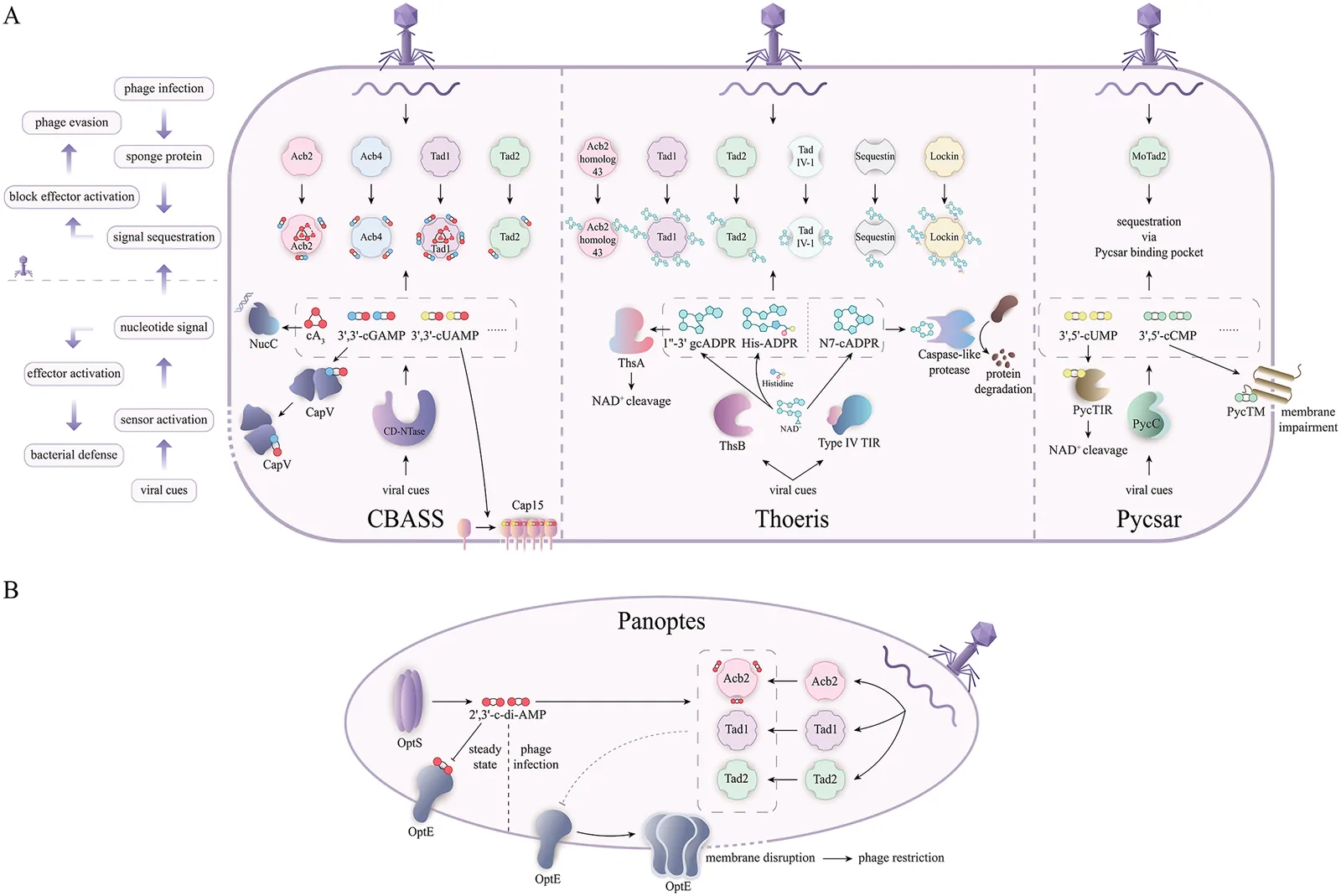
Overview of phage-encoded sponge proteins that broadly antagonize bacterial immune systems by sequestering signaling molecules. **(A)** Schematic overview of CBASS, Thoeris, and Pycsar immune signaling and their antagonism by phage-encoded sponge proteins. The shared logic is shown on the left, and the individual systems are detailed on the right. In the CBASS system, the CD-NTase sensor produces second messengers including 3′,3′-cGAMP, 3′,3′-cUAMP, and cA_3_, which activate CapV, Cap15, and NucC, respectively. The first two disrupt the membrane, whereas NucC degrades bacterial DNA. In the Thoeris system, ThsB converts NAD⁺ into 1′′-3′ gcADPR and, together with histidine, into His-ADPR. Both products stimulate ThsA to cleave NAD⁺, leading to NAD⁺ depletion. In type IV Thoeris, a TIR sensor generates N7-cADPR from NAD⁺, which activates a caspase-like protease that nonspecifically degrades cellular proteins. In the Pycsar system, PycC synthesizes cUMP and cCMP upon phage infection, activating downstream effectors including PycTIR (which cleaves NAD⁺) or PycTM (which disrupts the inner membrane), ultimately causing cell death or growth arrest. To counter these defenses, phages produce sponge proteins that sequester the corresponding signals. Acb2 broadly inhibits CBASS by binding three cyclic dinucleotides, including 3′,3′-cGAMP, and two cyclic trinucleotides such as cA_3_. Acb2 homologs, such as Acb2 homolog 43, sequester 3′cADPR to inhibit Thoeris. Acb4 binds nucleotide ligands including 3′,3′-cGAMP. Tad1 and Tad2 act across both systems. Tad1 has six peripheral pockets for cyclic dinucleotides and two central pockets for cyclic trinucleotides in CBASS, and binds six gcADPR molecules in Thoeris. Tad2 binds two cyclic dinucleotides in CBASS and two gcADPR molecules in Thoeris. Tad2 homologs such as MoTad2 bind Pycsar signals cUMP and cCMP. TadIV-1 specifically sequesters two N7-cADPR molecules. Two additional sponges are dedicated to Thoeris: Sequestin binds 1′′-3′ gcADPR; Lockin binds both 1′′-3′ gcADPR and His-ADPR, thereby inhibiting type I and type II Thoeris. Through these strategies, phages broadly suppress multiple bacterial immune systems. **(B)** Antagonism of the type I Panoptes immune system by phage-encoded sponge proteins. Under normal conditions, OptS constitutively generates cyclic dinucleotides such as 2′,3′-c-di-AMP, which bind the transmembrane effector OptE and keep it inactive. Upon phage infection, phage-encoded sponges including Acb2, Tad1, and Tad2 sequester these messengers, releasing OptE from inhibition. Freed from repression, OptE undergoes conformational changes and oligomerizes in the absence of the nucleotide, triggering disruption of the inner membrane and restricting phage replication, ultimately leading to cell death or growth arrest. Among these antagonists, Acb2 specifically targets 2′,3′-c-di-AMP in the type I Panoptes pathway.

CBASS systems are found in ~10% of sequenced bacterial genomes [14]. They are built around cGAS/DncV-like nucleotidyltransferases (CD-NTases), enzymes that generate diverse cyclic di- and trinucleotide signals upon recognition of phage infection [15]. These signaling molecules activate associated effector proteins (Caps), which execute growth arrest or cell death through membrane disruption, DNA degradation, or metabolite depletion (Fig 1A). For instance, CapV, the most abundant CBASS effector, is a phospholipase activated by 3′,3′-cGAMP that degrades membrane phospholipids [16]. Cap15 recognizes cyclic dinucleotides containing at least one uridine base, such as 3′,3′-cUAMP and c-di-UMP, and oligomerizes to disrupt the inner membrane [17]. NucC is an endonuclease activated by the cyclic trinucleotide cA_3_ that nonspecifically cleaves bacterial DNA (Fig 1A) [18].

Thoeris systems operate on a related principle. The ThsB sensor protein, via its Toll/interleukin-1 receptor (TIR) domain, synthesizes NAD⁺-derived signals, such as 1''–3' gcADPR in type I Thoeris [12] or His-ADPR in type II Thoeris [13]. These signals then bind to the ThsA effector, which cleaves NAD⁺, leading to NAD⁺ depletion and subsequent cell death or growth arrest. Separately, type IV Thoeris employs a distinct TIR-domain sensor that, upon phage infection, produces N7-cADPR to activate a caspase-like effector, which then nonspecifically degrades cellular proteins (Fig 1A).

Beyond these two systems, Pycsar, another cyclic nucleotide-based defense system, shares a similar signaling logic with CBASS and Thoeris and has been shown to be antagonized by phage-encoded sponges [7]. Upon phage infection, the pyrimidine cyclase PycC synthesizes cyclic pyrimidine mononucleotides as second messengers, including 3′,5′-cyclic UMP (cUMP) and 3′,5′-cyclic CMP (cCMP). These signals activate downstream effectors such as the TIR-domain effector PycTIR, which cleaves NAD⁺, or the transmembrane effector PycTM, which disrupts the inner membrane, ultimately triggering antiviral responses (Fig 1A) [19]. Type III CRISPR-Cas systems also employ nucleotide signaling. Upon phage infection, the Cas10 subunit synthesizes cyclic oligoadenylates (cOAs) as second messengers, which activate downstream effectors containing CARF or SAVED domains to execute antiviral responses [2].

## Phage countermeasures: Sponge proteins

As bacteria have refined their immune defenses, phages have evolved an array of counterstrategies. Some of these are anti-defense proteins that directly antagonize immune components. For instance, Acb1 and Apyc1 degrade cyclic nucleotide signals [3], while Tad3 physically blocks effector activation [20]. Particularly effective are sponge‑like proteins, small phage‑encoded polypeptides that sequester bacterial nucleotide signals without degrading them [21,22]. By depleting these signaling molecules, sponge proteins block effector activation and neutralize the host immune response, enabling phage replication [23]. To date, seven distinct families of phage-encoded sponge proteins have been experimentally characterized: Acb2, Acb4, Tad1, Tad2, Sequestin, Lockin, and TadIV-1 (Fig 1A) (Table 1) [4–6,8,9]. Despite their diverse amino acid sequences and oligomeric structures, these proteins share a common functional principle. They assemble into multimeric complexes that present deep, positively charged binding pockets capable of accommodating specific nucleotide ligands with varying degrees of promiscuity.

**Table 1:** Summary of phage-encoded sponge proteins and their nucleotide sequestering properties.

Sponge protein	Oligomeric state	Subunit composition	Binding sites/stoichiometry	Ligand specificity	Targeted systems	References
Acb2	Hexamer	Three dimers	2 CTN sites (central, at trimer interfaces) + 3 CDN sites (peripheral)	CDNs (e.g., 3',3'-cGAMP); CTNs (e.g., cA_3_)	CBASS	[4,7]
Acb2 homolog 43	Hexamer	Six protomers	2 sites (previously unobserved pockets, distinct from Acb2 CDN-binding site)	3'cADPR	Thoeris (type I)	[7]
Acb4	Tetramer	Four protomers	4 equivalent interfaces, each formed by three protomers	Diverse CDNs (e.g., 3',3'-cGAMP, 3',3'-cUA, 3',2'-cGAMP, 2',3'-cGAMP, 3',3'-cAA, 3',3'-cGG, 3',3'-cUU)	CBASS	[5]
Tad1	Hexamer	Three dimers	2 + 6 architecture (2 central CTN sites + 6 peripheral CDN/gcADPR sites)	CBASS: CTNs (e.g., cA_3_) and CDNs (e.g., 3',3'-cGAMP); Thoeris: gcADPR	CBASS, Thoeris	[24]
Tad2	Tetramer	Four protomers	2 + 2 architecture (2 CDN binding sites + 2 gcADPR binding sites)	CBASS: CDNs (e.g., 3',3'-cGAMP); Thoeris: gcADPR and His-ADPR	CBASS, Thoeris	[25]
MoTad2 (Tad2 homolog)	Tetramer	Four protomers	binds His-ADPR and cUMP/cCMP in distinct pockets	Thoeris type II: His-ADPR: Pycsar: cUMP, cCMP	Thoeris (type II), Pycsar	[7]
Sequestin	Dimer	Two protomers	2 sites (dimer interface)	1''-3' gcADPR	Thoeris (type I)	[9]
Lockin	Hexamer	Six protomers	6 sites (hexamer interfaces)	1''-3' gcADPR, His-ADPR	Thoeris (type I & II)	[9]
TadIV-1	Dimer	Two protomers	N-terminal flexible loop	N7-cADPR	Thoeris (type IV)	[6]

Acb2, for example, assembles into a hexamer composed of three dimers with two distinct binding sites that simultaneously capture cyclic dinucleotides (such as 3',3'-cGAMP) and cyclic trinucleotides (such as cA_3_), thereby antagonizing multiple CBASS subtypes at once (Fig 1A) [4,7]. Acb4, discovered as a founding member of a large family of immune evasion proteins, assembles into a tetramer with four equivalent binding interfaces that collectively sequester diverse ligands, such as 3′,3′-cGAMP and 3′,3′-cUA [5]. Tad1 [24] and Tad2 [25] are cross-system sponges that sequester signals from both Thoeris (gcADPR [8] and His-ADPR [13]) and CBASS [8], showing remarkable ligand-binding promiscuity (Fig 1A) [8]. Tad1 forms a hexamer composed of three dimers with a 2 + 6 binding architecture: two central pockets bind cyclic trinucleotides of CBASS, and six peripheral pockets accommodate cyclic dinucleotides of CBASS or gcADPR of Thoeris [8]. Tad2 forms a tetramer with a 2 + 2 architecture, in which two opposing binding interfaces each sequester either cyclic dinucleotides of CBASS or Thoeris signals such as gcADPR [8]. Sequestin and Lockin are dedicated Thoeris inhibitors that act as nucleotide sponges. Sequestin targets type I Thoeris by binding 1''-3' gcADPR, whereas Lockin inhibits both type I and type II Thoeris by binding 1''-3' gcADPR and His-ADPR, respectively [9]. TadIV-1, the most recently identified member, targets type IV Thoeris by binding N7-cADPR through a unique N-terminal flexible loop not observed in other sponge proteins [6].

Collectively, these findings illustrate the remarkable functional diversity of phage-encoded sponge proteins. Recent work has further revealed that some sponges exhibit even broader and less specific targeting capabilities than previously appreciated. Acb2 homologs, such as Acb2 homolog 43, use two previously unobserved pockets distinct from the Acb2 CDN-binding site to sequester 3′cADPR, thereby inhibiting Thoeris defense. Tad2 homologs, such as MoTad2, can bind the Pycsar cyclic mononucleotides cUMP and cCMP [7]. This expanded targeting repertoire suggests that the ability to sequester a wide range of nucleotide signals, rather than specifically targeting a single system, may serve as a key evolutionary advantage for phages.

## Bacterial counter‑countermeasures: The Panoptes system

If sponge proteins represented a clever phage solution to bacterial immunity, bacteria have recently answered with even more ingenious countermoves. Several antiphage systems detect phage evasion, including phage anti-restriction-induced system (PARIS), PrrC, and Hailong, which monitor viral proteins [26], restriction system status [27], and DNA integrity [28], respectively. PARIS and PrrC directly recognize anti-defense proteins, whereas the Hailong system employs a decoy single-stranded DNA (ssDNA) to detect phage infection. More recently, the newly discovered Panoptes system has joined this growing list. It stands out, however, by using nucleotide second messengers as the sensing cue, thereby inverting the logic of the arms race and adding a distinct twist [10,11,29].

Panoptes comprises at least two types. In type I, Panoptes is encoded by a two-gene operon, *optS* and *optE* (Fig 1B) [11]. OptS is a constitutively active minimal CRISPR polymerase (mCpol) that continuously produces cyclic dinucleotides, including 2′,3′-c-di-AMP, 3′,2′-cGAMP, and other related signals. OptE is a transmembrane effector protein containing two transmembrane helices and a soluble nucleotide-binding domain. Under steady-state conditions, the cyclic dinucleotides produced by OptS bind directly to the soluble domain of OptE, locking it in an inactive conformation and preventing its toxicity (Fig 1B) [11]. Type II Panoptes, which accounts for approximately 40% of all Panoptes instances, is encoded by a two-gene operon consisting of an mCpol cyclase and a CARF-TM effector [29]. The mCpol cyclase generates the cyclic trinucleotide cA_3_, which binds to the CARF-TM effector and holds it in an inactive dimeric state [29]. This arrangement is itself highly unusual. Most immune systems are activated by infection signals, not constitutively primed with repressive ligands that must be removed for activation.

The true novelty of Panoptes lies in what happens upon phage infection. When a phage infects the bacterial cell and deploys its sponge proteins to sequester nucleotide signals, an essential step in its immune evasion strategy, those sponges bind not only to their intended targets from CBASS and Thoeris but also to the cyclic dinucleotides produced by OptS in type I [11] or the cyclic trinucleotide cA_3_ produced by mCpol in type II [29]. In the process of sequestering these decoy signals, the sponges strip the inhibitory ligands away from OptE in type I [11] and CARF-TM in type II [29]. Liberated from repression, OptE undergoes conformational changes and oligomerizes in the absence of OptS-derived nucleotide, triggering disruption of the bacterial inner membrane and restriction of phage replication (Fig 1B) [10,11]. The CARF-TM effector in type II similarly drives oligomerization, ultimately leading to outer membrane permeabilization and growth arrest [29]. Overall, phage sequestration of nucleotides, normally an evasion tactic, becomes the trigger for host defense, inverting the logic of immune sensing.

Genetic and biochemical experiments have confirmed that in type I Panoptes, Acb2 directly sequesters 2′,3′-c-di-AMP and that this sequestration activity is sufficient to trigger activation [11]. Acb2 thus exhibits a dual function in the arms race. It helps phages evade CBASS and, in some cases, Thoeris, by sequestering nucleotide signals in one context, but in Panoptes-expressing bacteria, this very same sequestration activity is repurposed as a lethal trigger for cell death. For type II Panoptes, heterologous expression of Acb2 has similarly been shown to sequester cA_3_ and activate the CARF-TM effector, leading to cell toxicity [29]. Whether phage-encoded Acb2 activates type II Panoptes during natural infection remains to be demonstrated.

Panoptes activation is not limited to Acb2. In type I Panoptes, co-expression of Tad1 or Tad2 homologs with the OptSE operon also reduces bacterial colony formation (Fig 1B) [11]. This suggests that Panoptes responds to multiple phage-encoded antagonists that share the ability to deplete its cognate cyclic dinucleotide signals, including those primarily targeting Thoeris. Such broad-spectrum detection may reflect sensing of signal loss rather than recognition of the sponge proteins themselves. Consistent with this model, Apyc1 and AcrIII-1, which target cyclic mono- and tetranucleotides, do not activate Panoptes, whereas Acb1, a phosphodiesterase that degrades 2′,3′-c-di-AMP, does [11]. However, the precise determinants and the specific cyclic dinucleotides recognized by Tad proteins in the type I Panoptes context remain to be further characterized. Researchers have suggested that Tad1 and Tad2 likely activate Panoptes by binding and sequestering 2′,3′-c-di-AMP, the signaling molecule of the Panoptes system [11], but direct biochemical evidence for this mechanism is still lacking.

## Conclusions and prospects

Panoptes joins a growing list of antiphage systems, including PARIS [26], PrrC [27], and Hailong [28], that detect phage evasion. The Panoptes system reveals an extraordinary evolutionary twist: bacteria have converted a reliable phage evasion tactic into a deadly trap. Rather than detecting phages directly, Panoptes monitors the loss of cyclic nucleotides caused by phage-encoded antagonists, a loss that is itself a consequence of immune evasion. This decoy-sensing strategy adds to the known repertoire of indirect immune surveillance mechanisms in bacteria.

Several broader implications deserve mention. First, beyond the known examples of PARIS, PrrC, and Hailong, the decoy-based logic of Panoptes suggests that additional guardian or trap systems may exist and await discovery, monitoring various cellular metabolites, signaling molecules, or essential cellular processes for signs of phage interference. Systematic exploration of bacterial defense islands, known hotspots of immune gene clustering, may uncover additional systems that employ similar decoy-based logic. Second, the evolutionary connection between CBASS and the eukaryotic cGAS-STING pathway has prompted interest in whether higher organisms employ analogous strategies to detect viral immune antagonists. The guard/decoy model, in which a sensor protein monitors a host target or its mimic and triggers defense when a pathogen effector modifies either of them, is already well established in plant NLR immunity [30]. Recent work has also identified TIR-domain-containing proteins in higher organisms, including mammals, that produce signaling molecules reminiscent of bacterial Thoeris systems, suggesting that this immune logic may extend beyond plants [31]. Panoptes fits squarely within this guard/decoy framework, using decoy nucleotides to sense phage evasion. However, an important open question remains: no eukaryotic equivalent of phage-encoded sponge proteins has yet been identified. Whether such proteins exist in higher organisms, and whether they function through similar sequestration mechanisms, remains to be determined. Third, from a translational perspective, Panoptes offers conceptual inspiration for novel antiviral drug design and synthetic biology applications. Rather than directly targeting viral functions, an approach that often faces challenges with resistance development, one might instead exploit viral countermeasures and turn the pathogen’s own evasion strategies against it.

Several key questions define the frontier of this emerging field. How does oligomerization of OptE lead to membrane pore formation and cell death? Structural and biochemical studies are needed to determine the mechanism. What is the complete repertoire of phage-encoded antagonists that can trigger Panoptes activation, and what structural features determine their ability to sequester the cognate nucleotide signals? Does Panoptes exist in bacterial lineages beyond those currently identified, and does it cooperate or synergize with other immune systems within the same cell? Addressing these questions will deepen mechanistic insight and may uncover additional layers of the arms race.

In summary, Panoptes illustrates the sophistication of bacterial immunity and the evolutionary plasticity of host-pathogen interactions. By inverting phage evasion into a self-destruct signal, bacteria have gained a decisive, if temporary, advantage. As phages respond with new countermeasures, the cycle continues, promising more surprises. The discovery of Panoptes reminds us that the most effective biological solutions often come from understanding the rules of the game and then subverting them.

### Ethics statement

This work does not generate original data on humans or animals.

## References

[1] Brüssow H, Hendrix RW. Phage genomics: small is beautiful. Cell. 2002;108(1):13–6. doi: 10.1016/s0092-8674(01)00637-7 11792317

[2] Hobbs SJ, Kranzusch PJ. Nucleotide immune signaling in CBASS, Pycsar, Thoeris, and CRISPR antiphage defense. Annu Rev Microbiol. 2024;78(1):255–76. doi: 10.1146/annurev-micro-041222-024843 39083849 PMC12335278

[3] Hobbs SJ, Wein T, Lu A, Morehouse BR, Schnabel J, Leavitt A, et al. Phage anti-CBASS and anti-Pycsar nucleases subvert bacterial immunity. Nature. 2022;605(7910):522–6. doi: 10.1038/s41586-022-04716-y 35395152 PMC9117128

[4] Cao X, Xiao Y, Huiting E, Cao X, Li D, Ren J, et al. Phage anti-CBASS protein simultaneously sequesters cyclic trinucleotides and dinucleotides. Mol Cell. 2024;84(2):375-385.e7. doi: 10.1016/j.molcel.2023.11.026 38103556 PMC11102597

[5] Chang RB, Toyoda HC, Hobbs SJ, Hadary R, Richmond-Buccola D, Wein T, et al. A widespread family of viral sponge proteins reveals specific inhibition of nucleotide signals in anti-phage defense. Mol Cell. 2025;85(16):3151-3165.e6. doi: 10.1016/j.molcel.2025.07.016 40845805 PMC12380128

[6] Chen Y, Wei L, Han Z, Zhao R, Zeng Z, Liu Y, et al. A phage-encoded sponge protein suppresses bacterial TIR-Caspase immune signaling. Nat Commun. 2026;17(1):5047. doi: 10.1038/s41467-026-71527-4 41968157 PMC13243611

[7] Hadary R, Chang RB, Béchon N, Tal N, Osterman I, Yirmiya E, et al. Functional diversity of phage sponge proteins that sequester host immune signals. Nat Microbiol. 2026;11(6):1612–25. doi: 10.1038/s41564-026-02352-0 42230830

[8] Li D, Xiao Y, Fedorova I, Xiong W, Wang Y, Liu X, et al. Single phage proteins sequester signals from TIR and cGAS-like enzymes. Nature. 2024;635(8039):719–27. doi: 10.1038/s41586-024-08122-4 39478223 PMC12159963

[9] Tal N, Hadary R, Chang RB, Osterman I, Jacobson R, Yirmiya E, et al. Structural modeling reveals phage proteins that manipulate bacterial immune signaling. Science. 2026;391(6789):eaea1761. doi: 10.1126/science.aea1761 41785351

[10] Doherty EE, Adler BA, Yoon PH, Hsieh K, Loi K, Armbruster EG, et al. A miniature CRISPR-Cas10 enzyme confers immunity by inhibitory signalling. Nature. 2025;647(8091):997–1004. doi: 10.1038/s41586-025-09569-9 41034576 PMC12657230

[11] Sullivan AE, Nabhani A, Izrailevsky DS, Schinkel K, Hoffman CRK, Robbins LK, et al. The Panoptes system uses decoy cyclic nucleotides to defend against phage. Nature. 2025;647(8091):988–96. doi: 10.1038/s41586-025-09557-z 41034579 PMC12657218

[12] Tamulaitiene G, Sabonis D, Sasnauskas G, Ruksenaite A, Silanskas A, Avraham C, et al. Activation of Thoeris antiviral system via SIR2 effector filament assembly. Nature. 2024;627(8003):431–6. doi: 10.1038/s41586-024-07092-x 38383786

[13] Sabonis D, Avraham C, Chang RB, Lu A, Herbst E, Silanskas A, et al. TIR domains produce histidine-ADPR as an immune signal in bacteria. Nature. 2025;642(8067):467–73. doi: 10.1038/s41586-025-08930-2 40307559

[14] Millman A, Melamed S, Amitai G, Sorek R. Diversity and classification of cyclic-oligonucleotide-based anti-phage signalling systems. Nat Microbiol. 2020;5(12):1608–15. doi: 10.1038/s41564-020-0777-y 32839535 PMC7610970

[15] Whiteley AT, Eaglesham JB, de Oliveira Mann CC, Morehouse BR, Lowey B, Nieminen EA, et al. Bacterial cGAS-like enzymes synthesize diverse nucleotide signals. Nature. 2019;567(7747):194–9. doi: 10.1038/s41586-019-0953-5 30787435 PMC6544370

[16] Kong J, Wu W, Ke S, Zhou Z, Xia S, Chen J, et al. Molecular mechanisms of CBASS phospholipase effector CapV mediated membrane disruption. Nat Commun. 2025;16(1):8611. doi: 10.1038/s41467-025-63658-x 41022836 PMC12480259

[17] Duncan-Lowey B, McNamara-Bordewick NK, Tal N, Sorek R, Kranzusch PJ. Effector-mediated membrane disruption controls cell death in CBASS antiphage defense. Mol Cell. 2021;81(24):5039-5051.e5. doi: 10.1016/j.molcel.2021.10.020 34784509

[18] Lau RK, Ye Q, Birkholz EA, Berg KR, Patel L, Mathews IT, et al. Structure and mechanism of a cyclic trinucleotide-activated bacterial endonuclease mediating bacteriophage immunity. Mol Cell. 2020;77(4):723-733.e6. doi: 10.1016/j.molcel.2019.12.010 31932164 PMC7065454

[19] Tal N, Morehouse BR, Millman A, Stokar-Avihail A, Avraham C, Fedorenko T, et al. Cyclic CMP and cyclic UMP mediate bacterial immunity against phages. Cell. 2021;184(23):5728-5739.e16. doi: 10.1016/j.cell.2021.09.031 34644530 PMC9070634

[20] Yirmiya E, Hobbs SJ, Leavitt A, Osterman I, Avraham C, Hochhauser D, et al. Structure-guided discovery of viral proteins that inhibit host immunity. Cell. 2025;188(6):1681-1692.e17. doi: 10.1016/j.cell.2024.12.035 39855193

[21] Huiting E, Cao X, Ren J, Athukoralage JS, Luo Z, Silas S, et al. Bacteriophages inhibit and evade cGAS-like immune function in bacteria. Cell. 2023;186(4):864-876.e21. doi: 10.1016/j.cell.2022.12.041 36750095 PMC9975087

[22] Jenson JM, Li T, Du F, Ea C-K, Chen ZJ. Ubiquitin-like conjugation by bacterial cGAS enhances anti-phage defence. Nature. 2023;616(7956):326–31. doi: 10.1038/s41586-023-05862-7 36848932 PMC10097602

[23] Wang L, Zheng R, Zhang L. Sequestering survival: sponge-like proteins in phage evasion of bacterial immune defenses. Front Immunol. 2025;16:1545308. doi: 10.3389/fimmu.2025.1545308 40313938 PMC12043709

[24] Leavitt A, Yirmiya E, Amitai G, Lu A, Garb J, Herbst E, et al. Viruses inhibit TIR gcADPR signalling to overcome bacterial defence. Nature. 2022;611(7935):326–31. doi: 10.1038/s41586-022-05375-9 36174646

[25] Yirmiya E, Leavitt A, Lu A, Ragucci AE, Avraham C, Osterman I, et al. Phages overcome bacterial immunity via diverse anti-defence proteins. Nature. 2024;625(7994):352–9. doi: 10.1038/s41586-023-06869-w 37992756

[26] Deep A, Liang Q, Enustun E, Pogliano J, Corbett KD. Architecture and activation mechanism of the bacterial PARIS defence system. Nature. 2024;634(8033):432–9. doi: 10.1038/s41586-024-07772-8 39112702 PMC11479591

[27] Azam AH, Kondo K, Chihara K, Nakamura T, Ojima S, Nie W, et al. Evasion of antiviral bacterial immunity by phage tRNAs. Nat Commun. 2024;15(1):9586. doi: 10.1038/s41467-024-53789-y 39528469 PMC11555353

[28] Tan JMJ, Melamed S, Cofsky JC, Syangtan D, Hobbs SJ, Del Mármol J, et al. A DNA-gated molecular guard controls bacterial Hailong anti-phage defence. Nature. 2025;643(8072):794–800. doi: 10.1038/s41586-025-09058-z 40306316 PMC12360457

[29] Grüschow S, Wotherspoon P, Hilton-Balfe E, Graham S, White MF. Cyclic tri-adenylate controls a CARF-TM effector in type II Panoptes anti-phage systems. PLoS Biol. 2026;24(9):e3003934. doi: 10.1371/journal.pbio.3003934 42743106 PMC13577387

[30] Anbu S, Swart V, van den Berg N. Unmasking the invaders: NLR-mal function in plant defense. Front Plant Sci. 2023;14:1307294. doi: 10.3389/fpls.2023.1307294 38078096 PMC10698375

[31] Hurieva B, Osterman I, Falkovich AH, Yirmiya E, Leavitt A, Garb J. Conserved catalytic activity of immune TIR domains in animals. bioRxiv. 2026. doi: 10.64898/2026.04.20.719644

